# Impact of artificial intelligence on the training of general surgeons of the future: a scoping review of the advances and challenges

**DOI:** 10.1590/acb396224

**Published:** 2024-09-20

**Authors:** Caroliny Silva, Daniel Nascimento, Gabriela Gomes Dantas, Karoline Fonseca, Larissa Hespanhol, Amália Rego, Irami Araújo-Filho

**Affiliations:** 1 Universidade Federal do Rio Grande do Norte – General Surgery Department – Natal (RN) – Brazil.; 2 Universidade Federal de Campina Grande – General Surgery Department – Campina Grande (PB) – Brazil.; 3 Liga Contra o Câncer – Institute of Teaching, Research, and Innovation – Natal (RN) – Brazil.

**Keywords:** General Surgery, Specialties, Surgical, Artificial Intelligence, Education, Medical

## Abstract

**Purpose:**

To explore artificial intelligence’s impact on surgical education, highlighting its advantages and challenges.

**Methods:**

A comprehensive search across databases such as PubMed, Scopus, Scientific Electronic Library Online (SciELO), Embase, Web of Science, and Google Scholar was conducted to compile relevant studies.

**Results:**

Artificial intelligence offers several advantages in surgical training. It enables highly realistic simulation environments for the safe practice of complex procedures. Artificial intelligence provides personalized real-time feedback, improving trainees’ skills. It efficiently processes clinical data, enhancing diagnostics and surgical planning. Artificial intelligence-assisted surgeries promise precision and minimally invasive procedures. Challenges include data security, resistance to artificial intelligence adoption, and ethical considerations.

**Conclusions:**

Stricter policies and regulatory compliance are needed for data privacy. Addressing surgeons’ and educators’ reluctance to embrace artificial intelligence is crucial. Integrating artificial intelligence into curricula and providing ongoing training are vital. Ethical, bioethical, and legal aspects surrounding artificial intelligence demand attention. Establishing clear ethical guidelines, ensuring transparency, and implementing supervision and accountability are essential. As artificial intelligence evolves in surgical training, research and development remain crucial. Future studies should explore artificial intelligence-driven personalized training and monitor ethical and legal regulations. In summary, artificial intelligence is shaping the future of general surgeons, offering advanced simulations, personalized feedback, and improved patient care. However, addressing data security, adoption resistance, and ethical concerns is vital. Adapting curricula and providing continuous training are essential to maximize artificial intelligence’s potential, promoting ethical and safe surgery.

## Introduction

General surgery encompasses a variety of operative procedures, and learning evolves to meet the growing demands of modern medicine^1^. In recent years, remarkable progress has been observed in the application of artificial intelligence (AI) in the healthcare field, with a particular focus on diagnosis, personalized treatment, and clinical data analysis^2^^–^ 4.

The role of AI in surgical training is a topic of debate in the current context. The medical community, educators, and healthcare professionals urgently need to integrate AI into surgical training programs, while also dealing with ethical and practical issues that emerge in this transition process. This is evidenced by the growing body of scientific literature and the implementation of AI technologies in surgery residency programs worldwide^3^^–^ 5.

Smith et al.^1^ highlights that AI offers perspectives to improve surgical training, with the possibility of advanced simulations and intelligent assistance during procedures. However, ethical questions arise regarding liability in AI-assisted methods. Therefore, it is essential to understand how AI shapes surgical training and how we can balance such advances with its challenges, emphasizing the importance of an interdisciplinary dialogue to guide the ethical and effective integration of AI in general surgeons and other subspecialties training^6^^–^ 8.

Garcia et al.^2^ point out that one of the most promising aspects of AI in surgical training is the ability to create realistic simulations, allowing trainee surgeons to practice complex procedures in safe and controlled environment. However, Chen et al.^3^ warns of the challenging transition of AI-based surgical training, which involves the need for specialized teachers and access to relevant clinical data to feed learning algorithms in simulators. The importance of collaboration between medical institutions and technology companies to develop AI solutions that meet the specific needs of surgical training is emphasized. This collaboration highlights the relevance of the partnership between academia and industry in the search for advances in this area of health^9^^–^ 11.

In this context, AI emerges as an alternative that promises to shape the general surgeon of the future. This study explores the impact of AI on the training of these professionals, identifying advantages and challenges that permeate this educational and clinical revolution since the application of AI in general surgery has the potential to revolutionize medical practice^12^. However, the growing presence of AI in general surgeon training brings complex challenges. Brown and Jalali^4^ discussed the pressing need to address ethical issues such as accountability and transparency in AI-based clinical decision-making. Furthermore, Chen et al.^3^ highlights the importance of adequately integrating AI into the surgical training curriculum, ensuring that future surgeons are adapted to take advantage of the full potential of this technology.

Potential advantages of AI in general surgeon training include improving diagnostic accuracy, assistance during surgical procedures, and analysis of postoperative data^13^. In this same line, Rodriguez et al.^5^ highlight AI’s ability to provide real-time feedback during surgical training, accelerating the learning curve of trainee surgeons. However, the challenges of this transformation are crucial, such as ensuring that AI is used ethically and that its integration is effective in medical training, considering the long-term implications for surgical practice^14^^–^ 16.

In this sense, the objective of this review was to analyze the impact of AI on the training of the general surgeons of the future, exploring the potential advantages and challenges to be faced. We intend to incorporate scientific evidence that provides critical analysis for educators, healthcare professionals, and policymakers seeking to guide the surgery of the future in an environment increasingly influenced by technological advancement.

## Methods

This scoping review was conducted and reported in accordance with the Preferred Reporting Items for Systematic Reviews and Meta-Analyses extension for Scoping Reviews^17^. The research methodology involved a comprehensive search of multiple reputable databases to ensure the inclusion of relevant studies while minimizing the risk of bias. PubMed, Scopus, Scientific Electronic Library Online (SciELO), Embase, and Web of Science were chosen due to their comprehensive coverage of peer-reviewed literature in the medical field. Additionally, Google Scholar was utilized to access gray literature, which often includes valuable insights not found in traditional peer-reviewed articles. The selection criteria for the studies were centered on the study’s focus, which was AI’s impact on general surgeons’ training. To refine the search and capture relevant studies, a combination of keywords was used, including “general surgery,” “specialties, surgical,” “artificial intelligence,” “computational intelligence,” “computer reasoning,” and “education, medical.” This approach ensured that the selected studies were directly related to the topic of interest.

The inclusion criteria encompassed systematic reviews, case-control studies, cross-sectional studies, case series, review articles, and editorial studies. This broad inclusion criteria aimed to gather a comprehensive range of evidence and perspectives on the subject matter. The process of analysis, review, and selection of materials was conducted rigorously to maintain the quality and relevance of the chosen studies. All search records were imported into Rayyan for deduplication and revision by title/abstract.

In cases of disagreement between the two reviewers, a third reviewer was involved to reach a consensus and ensure the final selection of studies was based on well-founded criteria. This meticulous research methodology guarantees that the findings and conclusions drawn in the article are rooted in a robust and diverse body of evidence, enhancing the credibility and reliability of the study’s outcomes.

At least two reviewers independently conducted the extraction of the following data using a piloted extraction form: study design, setting, type of simulation, type of skill assessed, population, and purpose of using AI. Conflicts were adjudicated by a third reviewer. The quality assessment was conducted using the NewCastle-Ottawa quality assessment scale.

## Results and Discussion

**Figure 1:**
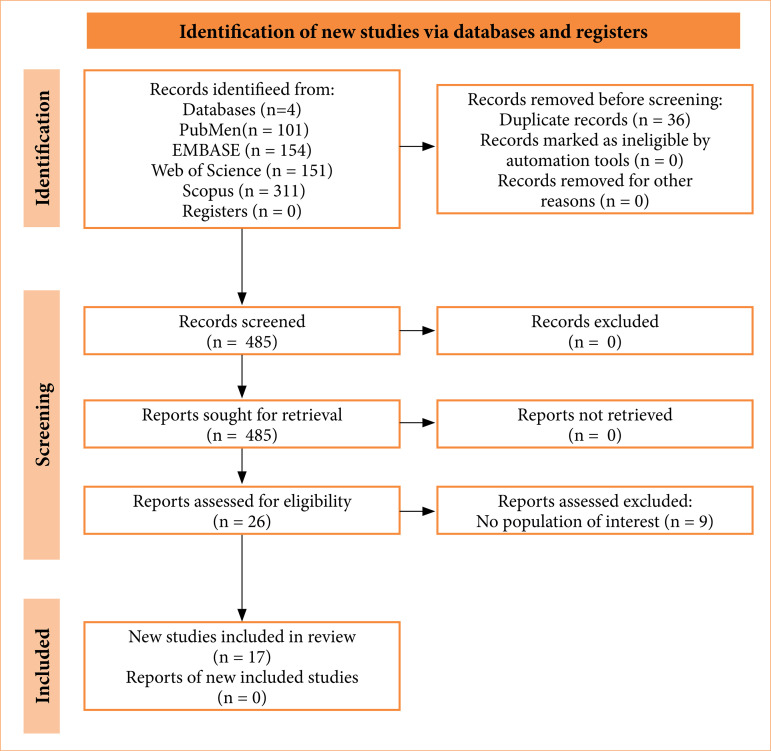
Identification of new studies via databases.

Source: Elaborated by the authors.

Screening results are presented in Fig. 1. From 581 studies reviewed, 17 were included in this scoping review, encompassing relevant reviews, experimental or observational studies. Table 1 summarizes the characteristics of the included studies, and Table 2 shows the quality assessment.

**Table 1:** Characteristics of the included studies.

Study	Design	Type of simulation	Type of skill assessed	Population	Purpose of using artificial intelligence	Levels of evidence*
Bissonnette et al.^18^	Comparative study	Virtual reality hemilaminectomy	Technical	41 (22 senior participants, six spine surgeons, three spine fellows, and 13 senior residents) and 19 junior participants (11 junior residents and eight medical students)	Assessment	3e
Brown et al.^19^	Comparative study	Peg Transfer	Technical	38 (13 fourth-year medical students, 14 surgical residents, four surgical fellows, and seven attending surgeons).	Assessment	3e
Chen et al.^20^	Comparative study	Laparoscopic assistance skills	Technical	74 (27 nurses, 31 clinical medical postgraduate students, and 16 residents)	Assessment, feedback	3e
Chmarra et al.^21^	Comparative study	Psychomotor laparoscopic skills	Technical	31 (10 experienced residents, 10 intermediates, and 11 novices)	Assessment	3e
Ershad et al.^22^	Comparative study	Endowrist manipulation, needle control and needle driving skills	Technical	14 (four experts practicing robotic surgical faculty, three surgical fellows post residency, three intermediates, and four medical students)	Assessment	3e
Frischknecht et al.^23^	Comparative study	Suturing performance	Technical	20 (five experts and 15 novices)	Assessment	3e
Kowalewski et al.^24^	Comparative study	Laparoscopic suturing and knot-tying	Technical	28 (eight novices, 10 intermediates, and 10 experts)	Assessment	3e
Kumar et al.^25^	Longitudinal study	Suturing, manipulation, transection, and dissection	Technical	Eight (six novices and two experts)	Assessment	2c
Loukas et al.^26^	Comparative study	Knot tying and needle driving	Technical	22 (experienced residents)	Assessment	3e
Loukas et al.^27^	Comparative study	Peg transfer and Knot-tying	Technical	32 (students and residents)	Assessment	3e
Mirchi et al.^28^	Comparative study	Brain tumor resection	Technical	50 (28 skilled and 22 novice)	Assessment	3e
Monserrat et al.^29^	Comparative study	Basic laparoscopic skills	Technical	15 (five high surgical knowledge, five intermediate surgical knowledge, and five novice surgical knowledge)	Assessment, feedback	3e
Pérez‐Escamirosa et al.^30^	Objective assessment	Psychomotor laparoscopic skills	Technical	43 (10 experienced surgeons and 33 non-experienced surgeons)	Assessment	3e
Riojas et al.^31^	Comparative study	Laparoscopic skills	Technical	38 (17 non-medical students, 11 medical students without previous laparoscopic surgery training, five medical students with some laparoscopic surgery training, four medical residents and one expert surgeon)	Assessment	3e
Saggio 2011	Comparative study	Skin pad incision, tissues dissection, interrupted stitch, running suture, knot-tying exercise	Technical	15 (five master surgeons, five resident surgeons and five attending surgeons)	Assessment	3e
Sgouros et al.^33^	Comparative study	Peg transfer and knot tying	Technical	74 (36 experts and 38 novices)	Assessment	3e
St John et al.^34^	Cross-sectional study	Artificial intelligence in diagnostic, operating room, medical management	Technical	31 (General surgery residents)	Assessment, feedback	4b

* According to Joanna Briggs Institute levels of evidence.

Source: Elaborated by the authors.

**Table 2:** Quality assessment of included studies

Study	Representative of the exposed cohort	Selection of external control	Ascertainment of exposure	Outcome of interest not present at the start of the study	Comparability of cohorts	Assessment of outcomes	Sufficient follow-up time	Adequacy of follow-up	Total (9/9)
Bissonnette et al.^18^	*	*	*	*	*	*	0	0	5/9
Brown et al.^19^	*	0	*	*	0	*	0	0	4/9
Chen et al.^20^	*	*	*	*	*	*	0	0	6/9
Chmarra et al.^21^	*	*	*	*	*	*	0	0	6/9
Ershad et al.^22^	NA	NA	NA	NA	NA	NA	NA	NA	NA
Frischknecht et al.^23^	*	0	*	*	0	*	0	0	4/9
Kowalewski et al.^24^	*	0	*	*	0	*	0	0	4/9
Kumar et al.^25^	*	0	*	*	0	*	0	0	4/9
Loukas et al.^26^	*	0	*	*	0	*	0	0	4/9
Loukas et al.^27^	*	0	*	*	0	*	0	0	4/9
Mirchi et al.^28^	*	0	*	*	0	*	0	0	4/9
Monserrat et al.^29^	NA	NA	NA	NA	NA	NA	NA	NA	NA
Pérez‐Escamirosa et al.^30^	*	0	*	*	0	*	*	*	5/9
Riojas et al.^31^	NA	NA	NA	NA	NA	NA	NA	NA	NA
Saggio 2011	NA	NA	NA	NA	NA	NA	NA	NA	NA
Sgouros et al.^33^	NA	NA	NA	NA	NA	NA	NA	NA	NA
St John et al.^34^	*	*	*	*	*	*	0	0	6/9

NA: Not applicable;;

* Source: elaborated by the authors.

The current medical landscape is being shaped by the technological revolution, with AI playing a central role in transforming the training of general surgeons^15^. This scoping review aims to explore the opportunities and challenges associated with incorporating AI into surgeon training, with a specific focus on general surgery. In this study, we deeply investigated the impact of AI on surgical training, addressing advantages, challenges, and future perspectives. In addition to the previously cited studies, an extensive review of the scientific literature reveals a wide range of valuable insights into this emerging topic^36^.

The incorporation of AI in the training of general surgeons represents a paradigmatic revolution in contemporary medical practice. The use of virtual surgical simulations, driven by advanced AI algorithms, provides an immersive and realistic training environment. These simulations allow trainee surgeons to practice complex procedures in a virtual environment, honing their technical skills before entering the operating room. This approach not only accelerates the learning curve, but also provides a safe space for experimentation and error without compromising patient safety.

Personalization of training is another notable benefit of AI integration. Algorithms can analyze individual performance, identify strengths and areas for improvement, and adapt training programs accordingly. This not only optimizes the use of training time, but also recognizes the diversity of skills among trainee surgeons. However, it is essential to address ethical issues such as over-reliance on algorithms in decision-making during surgical procedures. The transition to autonomous learning, although promising, must be carefully considered to ensure patient safety and the ethical integrity of medical practice.

Furthermore, AI can play a significant role in objectively assessing surgical competencies. Automated systems can analyze performance in detail, offering a more comprehensive and accurate assessment. However, it is essential to find a balance between the objectivity of algorithms and understanding the nuances inherent to surgical practice. Ethical and legal issues related to the use of AI in training, such as legal liability in cases of error, also require meticulous consideration. Establishing solid ethical guidelines and legal frameworks is crucial to ensuring that the implementation of AI in surgical training is ethical, transparent and accountable.

Zhang et al.^6^ emphasize that one of the main advantages of AI in surgical training is the creation of highly realistic simulation environments. This allows surgeons-in-training to practice complex procedures repeatedly, acquiring fundamental technical skills without putting patients at risk^6^^,^ 7. Furthermore, AI offers personalized feedback in real-time, identifying areas for improvement and precisely directing training, as Smith et al. pointed out^8^.

AI’s ability to process large volumes of clinical data, such as medical images and patient records, is undeniably valuable. Wang and Summers^9^ demonstrated that AI could identify patterns and trends that can be used to improve diagnoses and surgical planning, increasing surgeon effectiveness. Furthermore, Kim et al.^7^ highlight that AI has significant potential in performing assisted surgeries, in which surgical robots with AI can assist human surgeons, making procedures more precise and less invasive.

While AI offers many advantages, it also faces significant challenges. One of them is the issue of privacy and data security, as highlighted by Garcia et al^10^. The use of sensitive patient data in training future surgeons requires strict security policies in accordance with the regulations of each country or educational institution in which these surgeons are trained^19^.

Another challenge is resistance to adopting AI. Chen et al.^11^ noted that some surgeons and medical educators are reluctant to embrace AI out of fear that it may eventually replace their roles or that it will be unreliable. Overcoming this resistance requires efforts to raise awareness and demonstrate the benefits of AI in general surgery training and surgical subspecialties^37^^,^ 38.

It is essential to adapt the medical curriculum to optimize the use of AI in surgical training. Liu et al.^12^ emphasize that AI must be integrated from the beginning of training, ensuring that future surgeons fully understand its potential and limitations. This includes the introduction of specific subjects on AI and surgery^4^^–^ 43.

Furthermore, ongoing training is essential. Tang et al.^13^ highlights the importance of training programs that allow practicing surgeons to acquire skills in AI, ensuring that they can use it effectively in their clinical practices. In this way, the integration of AI into surgical training not only benefits surgeons, but also patients. Brown et al.^14^ points out that AI reduces medical errors, accurate diagnoses, and optimized surgical planning, resulting in safer and more effective procedures.

It is imperative to highlight the approach to ethical, bioethical, and legal issues involved in this entire process of technological incorporation, whether in surgery or any other area of health, as it involves human life^43^^–^ 45. Following this line of reasoning, Lee and Jeong^15^ highlight the need for clear guidelines for the ethical use of AI in surgery, ensuring transparency in clinical decisions. Adequate supervision and accountability are essential to avoid potential risks^15^^,^ 46.

As AI in surgical training continues to evolve, a continued focus on research and development is necessary. Future studies could further explore how AI can personalize training, tailoring it to the individual needs of trainee surgeons. Furthermore, it is essential to closely monitor the evolution of ethical and legal regulations related to the use of AI in medicine^47^^–^ 49.

## Conclusion

AI plays an increasingly significant role in training the general surgeon of the future. The advantages arising from technological advances are notable, which include advanced simulation, personalized feedback, clinical data analysis, and improvements in the quality of medical care. However, government control systems must seriously address challenges such as data security, resistance to adoption, and ethical and legal issues. Curricular adaptation and training are essential to ensure that surgeons make the most of AI in their clinical practices, resulting in more advanced, ethical, and safe general surgery.

## Data Availability

All data sets were generated or analyzed in the current study
